# Legislative Advisers

**Published:** 1920-03-06

**Authors:** 


THE PUBLIC WELL-BEING—*(continued)*

### Legislative Advisers.

Probably one of the most important practical women's organisations in this country is the little-known Joint Parliamentary Advisory Council, which comprises a body of eminent women experts on the different branches of social life and welfare questions, whose experience is brought to bear in a business-like way for the amendment, where necessary, of non-contentious Parliamentary measures. Lord Robert Cecil said at a meeting of the Committee a short time ago that the object of the Council was to get the women's point of view into the House of Commons. The need for this is clear, but it will be better still when the Advisory Council can give place to a Joint Parliamentary Council of Women Members of Parliament, but in all things progress must be made with the means at present to hand. This Council has been in existence since 1913, having been started by a group of Members of Parliament of various shades of political thought, who sent a letter to certain influential women, asking that such a Committee should be formed to keep in touch with the House of Commons and collect reliable information on all subjects connected with women and children. It has been considerably developed since then, and there are now various sub-committees to deal with separate questions. The Honorary Secretary, Mrs. Torrey, in explaining the functions of the Committee to a correspondent of the *Manchester Guardian*, said its work was in abeyance from the end of 1914 till 1915, when important amendments to the Children Bill were brought forward, but these have still to be dealt with, since much reform legislation lapsed during the war.

"Our housing amendments," she added, "only reached the Commons in time for the report stage, but a great many were accepted by the Lords, and by them referred to the Commons, who accepted them all. A very valuable report was that on the treatment and education of physically defective children. Our investigations showed how greatly these children were neglected in many cities, and we got that clause into the Education Act which makes it compulsory on local education committees to provide for their education. At the time we were told that if we persisted we should wreck the Education Bill because of the cost involved, so we felt it was a triumph when the amendment went through.

"There is no women's society doing just this sort of work, though no doubt they will develop it by degrees. My experience convinces me that the solid work women Members of Parliament will do in Committee will be of the greatest value. It has also shown the absurdity of scoffing at women's ability to tackle financial questions. I think that the very fact that they have generally been limited in the sums they have been able to spend on their households has made them keen on reducing expenditure and clever in making the most of their resources."

